# Safety and efficacy of photobiomodulation therapy in oncology: A systematic review

**DOI:** 10.1002/cam4.3582

**Published:** 2020-10-26

**Authors:** René‐Jean Bensadoun, Joel B. Epstein, Raj G. Nair, Andrei Barasch, Judith E. Raber‐Durlacher, Cesar Migliorati, Marie‐Thérèse Genot‐Klastersky, Nathaniel Treister, Praveen Arany, Joy Lodewijckx, Jolien Robijns, the World Association for Laser Therapy (WALT)

**Affiliations:** ^1^ Centre de Haute Energie Nice France; ^2^ City of Hope Comprehensive Cancer Center Duarte CA USA; ^3^ Cedars‐Sinai Health System Los Angeles CA USA; ^4^ Oral Medicine/Oral Oncology Griffith University and Haematology and Oncology Gold Coast University Hospital Queensland Health Gold Coast QLD Australia; ^5^ Harvard School of Dental Medicine Cambridge Health Alliance Cambridge MA USA; ^6^ Department of Oral Medicine Academic Centre for Dentistry Amsterdam University of Amsterdam and Vrije Universiteit Amsterdam Amsterdam The Netherlands; ^7^ Department of Oral and Maxillofacial Surgery Amsterdam UMC University of Amsterdam Amsterdam the Netherlands; ^8^ College of Dentistry Department of Oral and Maxillofacial Diagnostic Sciences University of Florida Gainesville FL USA; ^9^ Institut Jules‐Bordet Brussels Belgium; ^10^ Department of Oral Medicine Harvard School of Dental Medicine Boston MA USA; ^11^ School of Dental Medicine University of Buffalo Buffalo NY USA; ^12^ Faculty of Medicine and Life Sciences UHasselt Hasselt Belgium

**Keywords:** cancer, photobiomodulation, safety, supportive care, systematic review

## Abstract

We performed a systematic review of the current literature addressing the safety and efficacy of photobiomodulation therapy (PBMT) in cancer patients. In this systematic review, the Preferred Reporting Items for Systematic Reviews and Meta‐Analyses (PRISMA) guidelines were used. In vitro, in vivo, and clinical studies, which investigated the effect of PBMT on cell proliferation/differentiation, tumor growth, recurrence rate, and/or overall survival were included. The Medline/PubMed, EMBASE, and Scopus databases were searched through April 2020. A total of 67 studies met the inclusion criteria with 43 in vitro, 15 in vivo, and 9 clinical studies identified. In vitro studies investigating the effect of PBMT on a diverse range of cancer cell lines demonstrated conflicting results. This could be due to the differences in used parameters and the frequency of PBM applications. In vivo studies and clinical trials with a follow‐up period demonstrated that PBMT is safe with regards to tumor growth and patient advantage in the prevention and treatment of specific cancer therapy‐related complications. Current human studies, supported by most animal studies, show safety with PBMT using currently recommended clinical parameters, including in Head & Neck cancer (HNC) in the area of PBMT exposure. A significant and growing literature indicates that PBMT is safe and effective, and may even offer a benefit in patient overall survival. Nevertheless, continuing research is indicated to improve understanding and provide further elucidation of remaining questions regarding PBM use in oncology.

## Graphical abstract

We performed a systematic review of the current literature addressing the safety and efficacy of photobiomodulation therapy (PBMT) in cancer patients. A significant and growing literature indicates that PBMT is safe and effective, and may even offer a benefit in patient overall survival. Nevertheless, continuing research is indicated to improve understanding and provide further elucidation of remaining questions regarding PBM use in oncology.

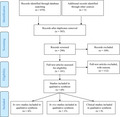

## 1 INTRODUCTION

In 1967, Dr. Endre Mester was the first scientist to discover that a low power laser had a stimulating effect on hair regrowth in mice.^1^ Since then, low‐level laser (LLL) has been applied for a variety of conditions and to boost physiological function in both humans and animals. In the past decade or so, the term photobiomodulation (PBM) replaced the former low‐level laser (LLL), and PBM was introduced as MESH word in PUBMED in 2015. Subsequently, the North American Association for Light Therapy (NAALT)^2^ and the World Association for Laser Therapy (WALT) defined photobiomodulation therapy (PBMT) as a form of light therapy that utilizes non‐ionizing forms of light sources, including laser diodes (LD), light‐emitting diodes (LEDs), and broadband light, in the visible and infrared spectrum. PBM provokes a nonthermal process whereby endogenous chromophores elicit photophysical and photochemical events at diverse biological levels. This process results in positive therapeutic outcomes including the stimulation of tissue regeneration and wound healing, the reduction of inflammation and pain, and immunomodulation.^3^

Since 1967, the number of clinical applications of light therapy has increased steadily in multiple medical fields, and in recent years PBM has been widely used for supportive care of cancer patients.^4^, ^5^ The best‐studied cancer therapy‐related complication, for which PBM is recommended, is oral mucositis (OM). The Mucositis Study Group of the Multinational Association of Supportive Care in Cancer/International Society for Oral Oncology (MASCC/ISOO) recommends the use of PBMT in the prevention of OM in patients with head and neck cancer undergoing chemoradiotherapy (CRT) and in stem cell transplant patients treated with high‐dose cytoreductive medications.^6^ PBM also has beneficial effects in the management of soft tissue necrosis in patients with head and neck cancer (HNC), and therapy‐induced bone necrosis.^7^, ^8^, ^9^ Also the potential application of PBM for management of xerostomia, dysgeusia, radiodermatitis, post‐RT fibrosis, chronic oral graft‐versus‐host disease (GVHD), and breast cancer‐related lymphedema has been reported.^10^, ^11^, ^12^, ^13^, ^14^, ^15^, ^16^, ^17^

The basic principle of supportive care in cancer is to provide effective management of complications of cancer treatment without compromising or inducing negative effects on oncology outcomes; also bearing in mind unwanted and dire consequences such as tumor persistence, new secondary tumors, or recurrence of the primary disease. Various in vitro studies have suggested that PBM may induce accelerated growth in some malignant cell lines and/or development of malignancy in dysplastic cells. Due to its increasing utilization in oncology care, and the better understanding of the biologic mechanisms and clinical outcomes, it is important to document the safety of PBM use in oncology settings. The aim of the present systematic review is to evaluate the available literature describing the safety and intervention outcomes in cancer patients receiving PBMT.

## 2 MATERIALS AND METHODS

### 2.1 Protocol

For this systematic review, the Preferred Reporting Items for Systematic Reviews and Meta‐Analyses (PRISMA) statement was used.^18^

### 2.2 Eligibility criteria

We considered for inclusion all in vivo and human clinical trials articles dealing with treatment and/or prevention of cancer therapy‐related complications, as well as in vitro studies on the safety of PBMT on cell lines. Case reports, cohort studies, case‐control studies, systematic and literature reviews, letters to the editor, theses, studies published in a language other than English, monographs, commentaries, conference abstracts, and unpublished data were excluded.

#### 2.2.1 Interventions

Studies that investigated the safety of PBM in an oncological setting, the potential tumor effects of PBM in cancer therapy, the mechanisms of PBM activity, molecular pathways, and differential effects upon tumor were reviewed. Robust safety data were desirable, considering the potential utility of PBM in supportive cancer care.

#### 2.2.2 Outcome measures

Mitotic rate, Tumor growth, Overall survival, Recurrence rate.

### 2.3 Information sources

We queried three databases (Medline/PubMed, EMBASE, and Scopus). To detect other potentially eligible reports that could meet the inclusion criteria, the reference list from all selected studies were checked by the reviewers. In addition, the included studies were screened to identify key authors. This allowed us for extra database searches based on author name. The last search was performed in April 2020.

### 2.4 Search strategy

Electronic searches were conducted in Medline/PubMed, EMBASE and Scopus using the following keywords alone or together: ("Laser Therapy"[Mesh] OR "Low‐Level light Therapy"[Mesh] OR "Laser Phototherapy"[Mesh] OR "Photobiomodulation Therapy"[Mesh] OR "Low‐intensity laser therapy"[Mesh] OR "Low‐Level Laser Irradiation"[Mesh]) AND ("cancer cell"[Mesh] OR "oncology"[Mesh] OR "tumor"[Mesh] OR "carcinoma"[Mesh] OR "neoplasm"[Mesh] OR "cancer"[Mesh] OR "radiotherapy"[Mesh] OR "chemotherapy"[Mesh] OR "oral mucositis"[Mesh] OR "radiodermatitis"[Mesh] OR "lymphedema"[Mesh] OR "dysgeusia"[Mesh] OR "xerostomia"[Mesh]) OR "hyposalivation"[Mesh] OR "trismus"[Mesh] OR "peripheral neuropathy"[Mesh] OR "osteoradionecrosis"[Mesh] AND "Overall survival"[Mesh] OR "Disease‐free survival"[Mesh] OR "Progression‐free survival"[Mesh] OR "proliferation"[Mesh] OR "apoptosis"[Mesh] OR "cell differentiation"[Mesh] OR "cell biology"[Mesh] OR "cell survival"[Mesh] OR "radiation effects"[Mesh]).

### 2.5 Study selection

All papers were systematically ordered in a Microsoft Office Excel 2016 document (Microsoft Corporation). The titles were checked and the duplicates excluded. Afterward, titles and abstracts were read for inclusion in the systematic review. Studies were classified into different categories: in vitro studies, in vivo studies, clinical studies, duplicates, no follow‐up/safety information, and language other than English. Two independent reviewers (RJB, JR) reviewed the studies assessed for eligibility in full‐text version. The studies lacking relevant methodological information were excluded.

### 2.6 Data extraction

Data from the included studies were extracted according to the following: (a) Author and publication year; (b) Study type (clinical trials and in vivo/in vitro);(c) PBM properties and treatment protocol; (d) type of animal models; (e) types of cells; (f) patient population; (g) duration of follow‐up; (h) outcome measures.

### 2.7 Data analysis

For this systematic review, a meta‐analysis was not feasible due to the great variation in PBM protocols used in the included studies. This systematic review presents a comprehensive qualitative synthesis of the results from the incorporated studies.

## 3 RESULTS

### 3.1 Study selection

**FIGURE 1:**
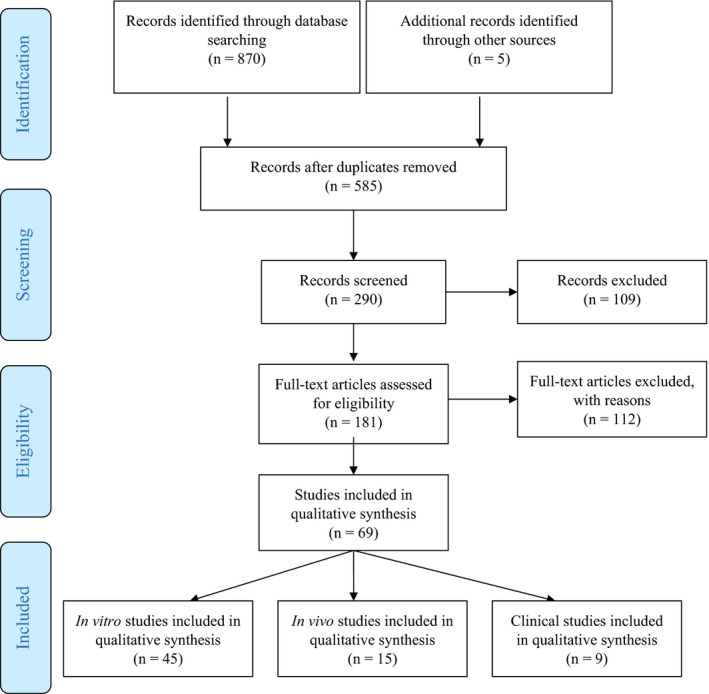
PRISMA flow diagram mapping out the number of records identified, included, and excluded

The flow diagram (Figure 1) gives on overview of the selection process of the included studies. In total, 870 studies were collected via the searches on the databases and 5 additional studies via the manual search. After a first review process, 585 duplicate studies were removed. One hundred and nine studies were excluded after reviewing the abstracts, and 114 studies were excluded as they did not meet the inclusion criteria. In total, 67 studies were included in the present systematic review: 43 in vitro, 15 in vivo, and 9 human clinical studies were analyzed.

### 3.2 Study characteristics

The study characteristics of the in vitro, in vivo, and human clinical studies are presented in Tables 1, 2, and 3, respectively.

**TABLE 1:** Study characteristics of the in vitro studies investigating the effect of PBMT on cancer cell lines

Author (Ref.)	Year	Cell type	PBM device	Wavelength	Fluence	Exposition time (sec)	Application protocol	Cell viability /proliferation
Marchesini^61^	1989	Colon carcinoma (HT29), Breast carcinoma (MCF7), Malignant melanoma (M14 and JR1)	Argon LD	N.S	4.2 and 150 kJ/m^2^	N.S.	Single application	Increases tumor cell culture growth
Tsai^50^	1991	Glioma cell (C6)	four different types: ‐ CO2 ‐ Argon ‐ HeNe ‐ GaAs	‐ 488–512 nm ‐ 632.8 nm ‐ 904 nm	‐ 0.4–22 J/cm^2^ ‐ 1.1–11 J/cm^2^ ‐ 2.7–326 mJ/cm^2^ ‐ 9–380 mJ/cm^2^	‐ 0.1 to 20 s ‐ 0.5 to 5 s ‐ 1 to 120 s ‐ 9 to 350 s	Single application	‐ He‐Ne laser induced a dose‐related biostimulatory effect ‐No dose related biostimulatory effect was noted after GaAS laser irradiation
Schaffer^49^	1997	Human squamous carcinoma cell lines of the gingival mucosa (ZMK)	LD	805 nm	2–20 J/cm^2^	N.S.	Single application	ZMK cells showed a decreased of mitotic index at 4 and 20 J/cm^2^
Sroka^51^	1999	Skeletal myotubes (C2), normal urothelial cells (HCV29), human squamous carcinoma cells of the gingival mucosa (ZMK1), urothelial carcinoma cells (J82), glioblastoma cells (U373MG), and breast adenocarcinoma cells (MCF7)	‐Kr+‐laser ‐ Ar+‐laser ‐ Ar+‐pumped tunable dye ‐GaAlAs‐LD ‐Nd:YAG laser	‐ 410 nm ‐ 630 nm ‐ 635 nm ‐ 640 nm ‐ 805 nm ‐ 1064 nm	0–20 J/cm^2^	N.S.	Single application	Increased mitotic rate for J82, HCV29 with 410, 635 and 805 nm; C2 with 635 nm Max mitotic rate: J82, HCV29, C2 with 4 and 8 J/cm^2^ Min mitotic rate: J82, HCV29, C2 with 20 J/cm^2^ Min mitotic rate for MCF7, U373MG, and ZMK1 with increasing J/cm^2^; All cell lines with 20 J/cm^2^
Coombe^59^	2001	Human osteosarcoma cell line, (SAOS−2)	GaAlAs LD	830 nm	1.7 to 25.1 J/cm^2^	N.S.	A single or daily irradiation for a period of 1–10 days	Cellular proliferation or activation was significantly influenced by any of the PBM parameters applied
Pinheiro^48^	2002	H.Ep.2 cells (SCC type 2)	LD	635‐ or 670‐nm	0.04, 0.06, 0.08, 1.2, 2.4, and 4.8 J/cm^2^	N.S.	7 consecutive days at the same daytime	PBM (670 nm) at a dose between 0.04 and 4.8 J/cm^2^ significantly increased proliferation of H.Ep.2 cells
Kreisler^52^	2003	Epithelial tumor cells from laryngeal carcinoma	GaAlAs‐LD	809 nm	1.96, 3.92, and 7.84 J/cm^2^	75, 150, 300 s	Single application	The irradiated cells demonstrated a higher proliferation rate up to 3 days post‐PBM
Liu^61^	2004	Human hepatoma cell line (HepG2 and J−5 cells)	GaAlAs‐LD	808 nm	5.85 and 7.8 J/cm^2^	90, 120 s	Single application	PBM inhibited the proliferation of HepG2 and J−5 cells
Mognato^53^	2004	Human epithelial adenocarcinoma (HeLA) and lymphoblast cell line (TK6)	In‐Ga‐As LD	808–905 nm	1, 4, 15, 30, and 60 J/cm^2^	N.S.	Single application	PBM did not affect HeLa cells at 808 nm but stimulated proliferation at 905 nm and combined wavelengths. TK6 cells were not affected.
Werneck^55^	2005	H.Ep.2 cells (human SCC larynx)	LD	685 nm 830 nm	4 J/cm^2^	N.S.	Single application	PBM improved cellular proliferation in cells at 685 nm or 830 nm wavelengths. Top proliferation was detected at 12 h (685 nm) and at 6 h and 48 h (830 nm).
De Castro^54^	2005	Human oral carcinoma cells^101^	LD	685 nm 830 nm	4 J/cm^2^	N.S.	One or two applications	PBM (830 nm) increased proliferation at 12 h. The increase was noticeable up to 48 h. No response in cells treated with PBM at 685 nm.
Liu^56^	2006	Human hepatoma cell line (HepG2 and J−5 cells)	GaAlAs‐LD	808 nm	0, 1.95, 3.9, 5.85, 7.8, 9.75, and 11.7 J/cm^2^	0, 30, 60, 90, 120, 150, and 180 s	Single application	PBM at 5.85 and 7.8 J/cm^2^ inhibited the survival of human HepG2 cells
Renno^57^	2007	Human osteosarcoma cell line (MG63)	LD	830 nm 780 nm 670 nm	0.5, 1, 5, 10 J/cm^2^	N.S.	Single application	PBM at 670 nm increased osteosarcoma cell proliferation significantly (at 5 J/cm^2^). The same was true at 780 nm laser (at 1, 5, and 10 J/cm^2^), but not after 830 nm PBM
Powell^58^	2010	Human breast cancer cell line (MCF−7 – adenocarcinoma) and a human melanoma cell line (MDA‐MB−435S/M14)	GaAlAs‐LD	780 nm 830 nm 904 nm	0.5, 1, 2, 3, 4, 10, 12, and 15 J/cm^2^		1–3 applications with 24 h in between	Minimal changes were detected in the growth rates of MDA‐MB−435S (melanoma) cells after a single PBM treatment, regardless of the PBM parameters applied. Increased proliferation of MCF−7 with 1, 2, 4, 10, and 12 J/cm^2^ at 780 nm and at 0.5, 1, 3, 4 and 15 at 904 nm
Huang^47^	2011	ASTC‐a−1 cells, HeLa cells, human hepatocellular liver carcinoma (HepG2) cells, and African green monkey SV−40‐transformed kidney fibroblast (COS−7)	HeNe LD	632.8 nm	20, 40, 80, 120, and 160 J/ cm^2^	1.66, 3.33, 6.66, 10, 13.33 min	Single application	PBM increased apoptosis via inactivation of the Akt/GSK3b signaling pathway through ROS production.
Al‐Watban ^46^	2012	Murine fibrosarcoma (RIF−1) Mouse mammary adenocarcinoma (EMT−6)	HeNe LD	632.8 nm	60, 120, 180, 240, 300, 360, 420, 480, 540, and 600 mJ/cm^2^	16, 32, 48, 64, 80, 96, 112, 128, 144, and 160 s	Three consecutive days	A trend of stimulation, zero‐bioactivation, and inhibition in all cell lines. The ideal biostimulatory dose was 180 mJ/cm^2^ and bio‐inhibitory doses were from 420–600 mJ/cm^2^ increasing doses.
Schartinger^45^	2012	Human oral SCC cell line (SCC−25)	GaAlAs‐LD	660 nm	N.S.	15 min	Three consecutive days	PBM led to an increase in the percentage of S‐ phase cells and a decrease in the percentage of G1‐phase cells. PBM induced a pro‐apoptotic effect and no tumor promoting effect.
Magrini^44^	2012	Human malignant breast cells (MCF−7)	HeNe LD	633 nm	5, 28.8, and 1000 mJ/cm^2^	1–16.5 min	Single application	PBM influenced cell metabolism and viability, depending on the fluence, for at least 6.5 days. PBM at 5 mJ∕cm^2^, had a bio‐inhibitory effect, which led to a decrease in cell metabolism. At 28.8 mJ∕cm^2^, no proliferation was detected, but there was an increase of the cell metabolism. At 1 J∕cm^2^, PBM led to an increase of cell metabolism.
Murayama^43^	2012	Human A−172 glioblastoma cell line	LD	808 nm	8, 36, and 54 J/cm^2^	20, 40, and 60 min	Single application	Suppressed proliferation in a fluence‐dependent manner
Sperandio^42^	2013	Human dysplastic oral keratinocytes (DOK cell line) Human oral squamous cell carcinoma cell lines (SCC9 and SCC25)	LD	660 nm 780 nm	0, 2.05, 3.07, and 6.15 J/cm^2^	N.S.	Single application	PBM changed growth of both cell lines by modulating the Akt/mTOR/CyclinD1 signaling pathway, both up regulating and down regulating depending on the used PBM parameters.
Basso^41^	2014	Osteosarcoma (Saos2)	InGaAsP LD	780 nm	0.5, 1.5, 3, 5, and 7 J/cm^2^	40, 120, 240, 400, and 560 s	Single application	PBM at 0.5 J/cm^2^ increased cell viability
Gomes Henriques^40^	2014	Human oral squamous cell carcinoma cell lines (SCC25)	InGaAsP LD	660 nm	0, 0.5, 1 J/cm^2^	16 and 33 s	Two applications, 48 hours in between	PBM significantly increased proliferation of SCC25 cells at 1.0 J/cm^2^.
Matsumoto^39^	2014	Human Colon cancer cell lines (HT29 and HCT116)	LED	465 nm 525 nm 635 nm	N.S.	10 min	Every 24 h for 5 days	PBM at 465 nm reduced viability of HT29 and HCT116 cells. However, PBM did not change viability of HT29 cells at 525 nm or 635 nm.
Tsai^68^	2015	Human osteosarcoma cell line (MG−63)	LD	810 nm	1.5 J/cm^2^	80 s	Single application before PDT	PBM increases the effect NPe6‐mediated photodynamic therapy via increased ATP synthesis.
Obayashi ^38^	2015	Pancreatic carcinoma cell line (KP4, PK−9, MIA‐PaCa2)	GaAlAs‐LD	915 nm	N.S.	3, 5, or 7 min	Single application	Upregulated apoptosis with increasing power and duration of irradiation
Cialdai^37^	2015	Human breast carcinoma cell lines (MCF−7 and MDA‐MB361)	LD	808 nm 905 nm	9 J/cm^2^	10 min	Three consecutive days with	PBMT did not significantly impact the behavior of human breast adenocarcinoma cells, including their clonogenic efficiency
Dastanpour^36^	2015	Acute myeloid leukemia (AML) cell line (KG−1a)	LD	810 nm	5, 10, and 20 J/cm^2^	N.S	One to three applications with 48 h in between	PBM significantly increase cell proliferation after two PBM exposures at an energy density of 20 J/cm^2^. Other PBM parameters did not affect cell proliferation.
Crous & Abrahamse ^70^	2016	Lung cancer stem cells (CSC) isolated from lung cancer cells (A549)	LD	636 nm	5, 10, and 20 J/cm^2^	8 min 54 s 17 min 48 s 35 min 36 s	Single application	PBM increased the cell density due to stimulation of cell proliferation
Ramos Silva^34^	2016	Human breast cancer cell line (MDA‐MB−231 cells)	GaAlAs LD	660 nm	30, 90, 150 J/cm^2^	30, 90, 150 s	Single application	PBM did not influence cell viability. PBM enhanced cell populations in S and G2/M cell cycle phases. PBM led to a decrease in proliferation and increase in senescence.
Barasch^33^	2016	Normal human lymphoblasts (TK6) Human leukemia cells (HL60)	HeNe LD	632.8 nm	0.1, 1, 2, 4, 8,12 J/cm^2^	3, 29, 57, 114, 229, 343 s	Single application	Pre‐radiation exposure to PBM (4.0 J/cm^2^) followed by 1‐h incubation hindered growth regression in TK6 but not in HL60 cells. PBM made the HL60 cells more susceptible to the killing effects of RT in a dose‐dependent way. Furthermore, exposure of HL60 to PBM alone led to cell death in a dose‐dependent way.
Schalch^32^	2016	Human lingual squamous cell carcinoma (SCC9)	LD	660 nm 780 nm	2.71, 5.43, 8.14 J/cm^2^	12.7, 25.3, 38 s	Single application	PBM of SCC9 cells (4 J/cm^2^) decreased the pro‐osteoclastogenic potential.
Kara^31^	2017	Saos−2 osteoblast‐like cells (ATCC85‐HTB) Human lung carcinoma cells (A549)	Nd:YAG laser	1064 nm	N.S.	0.5 min	Single application	PBM increased cancer cell proliferation, depending on the applied PBM parameters.
Djavid ^30^	2017	Human cervix adenocarcinoma cell line (HeLa)	LD	685 nm	0, 5, 10, 20 J/cm^2^	N.S.	Single application	PBM at different energy densities (5–20 J/cm^2^) was not cytotoxic. However, HeLa cells pre‐exposed to 20 J/cm^2^ showed improved inhibition of colony formation following RT. Enhanced radiosensitivity was related to more DNA damage, and oxidative stress, and radiation‐induced apoptosis and autophagy,
Bamps^29^	2018	Head and neck cancer (HNSCC) cell lines (SCC154, SQD9, and SCC61)	AsGaAl LD	830 nm	1–2 J/cm^2^	N.S.	Single application	PBM increased cell proliferation of HNSCC cell lines at 1 J/cm^2^, while no significant increase was seen after PBM at 2 J/cm^2^.
Schalch^28^	2018	Head and neck cancer (HNSCC) cell line (SCC9)	LD	660 nm 780 nm	1–6 J/cm^2^	8.4, 16.9, 12.7, 25.3, 38 s	Single application	PBM reduced mitochondrial activity in the SCC9 cells using 11 diverse PBM parameters. PBM at 780 nm (4 J/cm^2^) was the safest and led to a reduction in cell viability, the induction of apoptosis, and a reduction in the migration capacity of the cancer cells.
Diniz^27^	2019	Oral keratinocytes (HaCat) Tongue squamous cell carcinoma cells (SCC25) Upper aerodigestive tract carcinoma cells (HN12)	GaAlAs LD	660 nm	11.7 J/cm^2^	6 s	Single application	PBM led to an increase in sensitivity to cisplatin. PBM could potentiate the effects of cisplatin, leading to increased drug cytotoxicity and enhanced apoptosis.
Chen^26^	2019	Melanoma cells (B16F10 melanoma cells)	LED	418 nm 457 nm 630 nm	0.04,0.07,0.15, 0.22, 0.30, 0.37, 0.45, 0.56, 1.12	0, 450, 900, 1800 s	Single application	PBM at 418–457 nm inhibited the growth of the B16F10 melanoma cells and a high energy density had better results.
Takemoto ^25^	2019	Human OSCC cell line (CAL27)	LED	660 nm	3, 6 J/cm^2^, 9, 12, 24, and 36 J/cm^2^	N.S.	Three applications	PBM at high doses hindered the progression and number of OSCC colonies without affecting the surrounding stromal fibroblasts.
Levchenko^24^	2019	HeLa cells	LD	808 nm	0.3, 3, 10, and 30 J/cm^2^	6, 60, 200, and 600 s	Single application	PBM (0.3, 3, and 30 J/cm^2^) induced apoptosis along a gradual increase over time, in contrast to non‐irradiated cells and cells irradiated at 10 J/cm^2^
Matsuo^23^	2019	Squamous cell carcinoma cell line (HSC−3)	LED	630 nm	N.S.	N.S	Single application	PBM increased the migration ability of HSC−3 cells
Kianmehr^22^	2019	HDF cell line Human melanoma cancer cell lines (A375 and SK‐MEL−37)	LD	660 nm	3 J/cm^2^	90 s	Single application	PBM alone is not able to destroy human normal fibroblast and human melanoma cancer cells. PBM in combination with p‐Coumaric acid did not alter the cell viability in human fibroblasts but reduced the cell viability in melanoma cells probably via the apoptosis pathway.
Abuelmakarem^21^	2019	Colon cancer cell line (Caco−2 cell line)	LD	660 nm	N.S.	5 min	Single application	PBM decreased the cell viability.
Kiro^64^	2019	Isolated CSCs adenocarcinoma MCF7	LD	636 nm 825 nm 1060 nm	5, 10, 20, 40 J/cm^2^	10 min 48 s 20 min 9 s 40 min 21 s 1 h 20 min 30 s	Single application	PBM increased the cell proliferation and viability of BCCs and BCSCs after being exposed to 5–40 J/cm^2^ using wavelengths of 636, 825 and 1060 nm. PBM decreased cytotoxicity in both BCCs and BCSCs after treatment with low energy densities.
Khorsandi^20^	2020	Breast cancer cell lines (MDA‐MB−231) Melanoma cancer cell line (A375) Human dermal fibroblast cell line (HDF)	LD	660 nm	3 J/cm^2^	90 s	Single application	PBM alone cannot induce cell death in human normal and cancerous cells. PBM in combination with gallic acid (GA) treatment did not alter the cell viability in human normal cells but significantly reduced the survival of cancer cells more than GA alone.
Shakibaie^19^	2020	Breast cancer cell lines (MCF−7)	LED	435 and 629 nm	7.9 and 17.5 J/cm^2^	N.S.	Single application	PBM (435 nm) decreased the proliferation and metabolic activity of MCF−7 cells. PBM (626 nm) increased the metabolic activity and proliferation of MCF−7 cells.

Abbreviations: BCC, breast cancer cell; CSC, cancer stem cell; HNC, head and neck; LD, laser diode; LED, light emitting diode; PBMT, photobiomodulation therapy; ROS, reactive oxygen species; SCC, squamous cancer cell.

**TABLE 2:** Study characteristics of the in vivo studies investigating the effect of PBMT on cancer animal models

Author (Ref.)	Year	Animal type	Tumor type	PBM device	Wavelength	Fluence	Exposition time (sec)	Application protocol	Tumor growth rate/Tumorigenicity
Mikhailov^82^	1993	Rat	Walker's carcinosarcoma Cancer of the mammary gland (RMK−1)	LD	890 nm	0.46, 1.53 J/cm^2^	15 s	Five applications on consecutive days directly on the tumor	PBM at 0.46 J/cm^2^ led to retardation in tumor growth and life span was prolonged versus control animals. PBM increased dystrophic and necrotic changes in the tumor. Tumor weight increased at 1.53 J/cm^2^.
Abe^81^	1993	Mouse	Glioma	GaAlAs LD	830 nm	N.S.	15 s	Two applications/day, one day post implantation and Two applications/day, 14 days post implantation directly on the skin over the tumor site or indirect on the abdominal skin	PBM applied on the first day after glioma implantation, both in a direct and indirect manner, inhibited the tumor growth. At 14 days postimplantation indirect PBM enhanced tumor growth.
Ulrich^71^	1996	Rat	Rhabdomyosarcomas (R1H)	LD	830 nm	1 and 100 J/cm^2^	N.S.	15 fractions over 3 weeks	Single doses PBM do not inhibit nor stimulate tumor growth. Fractionated PBM does not alter growth kinetics of the tumors. Increase in tumor necrosis after 15 fractions of 100 J/cm^2^
Frigo^62^	2009	Mouse	Melanoma cell line (B16F10)	LD	660 nm	150 J/cm^2^ 1050 J/cm^2^	60 s 420 s	Once a day for three consecutive days	PBM at 150 J/cm^2^ was safe with only negligible effects on cell proliferation in vitro and no significant effect on tumor growth in vivo. PBM at a high irradiance (2.5 W/cm^2^) combined with high dose of 1050 J/cm^2^, could stimulate melanoma tumor growth.
Zhang^79^	2009	Mouse	Human cervical carcinoma cell line (HeLa)	LED	650 nm	N.S	N.S.	Single application	PBM diminished the tumor growth of tumors on day 50 and weakened the elevation of vascular endothelial growth factor (VEGF). PBM could induce HeLa cell apoptosis and have antitumor properties.
Monteiro^76^	2011	Hamster	Squamous cell carcinoma (SCC)	LD	660 nm	56.4 J/cm^2^	133 s	Every other day for 4 weeks	PBM led to a significant progression of the severity of SCC
Myakishev‐Rempel^77^	2012	Mouse	UV‐induced skin cancer	GaAlAs LED	760 nm	2.5 J/cm^2^	312 s	Twice daily for 37 days	PBM did not have an effect on the growth of the UV‐induced skin cancer.
Monteiro^78^	2013	Hamster	Squamous cell carcinoma (SCC)	LD	660 nm	95 J/cm^2^	133 s	Every other day for 4 weeks	PBM did not influence tumor behavior, four weeks after tumor induction.
Wikramanayake^83^	2013	Rat	Chemotherapy induced alopecia	LD	655 nm	N.S.	60 s	Daily for 10 days	PBM did not affect the efficacy of chemotherapy
Ottaviani^63^	2016	Mouse	Melanoma cell line (B16F10)	InGaAlAsP LD	660 nm 800 nm 970 nm	3 or 6 J/cm^2^	30–60 s	Once a day for 4 consecutive days	PBM hindered tumor progression, provoked tumor vessel normalization and stimulated the immune system to produce type I interferons.
Rhee^75^	2016	Mouse	Human anaplastic thyroid cell line (FRO)	LD	650 nm	15, 30 J/cm^2^	150 and 300 s	Single application	PBM decreased TGF‐β1 and increased p‐Akt/HIF−1α which resulted in proliferation and angiogenesis of anaplastic thyroid carcinoma (ATC)
Khori ^74^	2016	Mouse	Mouse mammary carcinoma (4T1, ATCC CRL−2539)	LD	405, 532, and 632 nm	N.S	N.S	10 treatments three times a week with a weekend break	PBM (405–532 nm) significantly reduced the tumor size.
Petrellis^73^	2017	Rat	Walker's carcinosarcoma	LD	660 nm	35.7, 107.14, 214.28 J/cm^2^	10 s 30 s 60 s	Three times on alternate days	PBM increased inflammatory markers IL−1β, COX−2, iNOS. PBM decreased inflammatory markers IL−6, IL−10, and TNF‐α. PBM at 1 J−35,7 J/cm^2^ produced cytotoxic effects by ROS generation.
Frigo^62^	2018	Mouse	Melanoma cell line (B16F10)	InGaAlP LD	660 nm	150, 450, 1050 J/cm^2^	60 s 180 s 420 s	Each 24 h for three consecutive days	High PBM doses (≥ 9 J) showed a dose‐dependent tumor growth, different collagen fibers characteristics, and eventually blood vessel growth. A PBM dose of 3 J did not affect the melanoma cell activity.
Barasch^72^	2019	Mouse	Human squamous cell carcinoma of the oral tongue (Cal−33)	LD	660 nm 850 nm	18.4 J/cm^2^ 3.4 J/cm^2^	75 s	(1) PBM at 660 nm, 18.4 J/cm^2^, and 5 RT ×4 Gy doses delivered daily; (2) PBM at 660 nm, 18.4 J/cm^2^, and 1 × 15 Gy RT; (3) PBM at 660 nm +850 nm, 45 mW/cm^2^, 3.4 J/cm^2^, and 1 × 15 Gy RT	RT‐treated animals survived significantly longer and had significantly smaller tumor volume when matched with the control and PBM treatment groups. No significant differences were discovered between the RT alone and PBM +RT groups in any of the experiments.

Abbreviations: LD, laser diode; LED, light emitting diode; PBMT, photobiomodulation therapy; ROS, reactive oxygen species; SCC, squamous cancer cell.

**TABLE 3:** Study characteristics of the clinical trials investigating the safety of PBMT in patients with cancer

Author (Ref.)	Year	Type of study	Patient type	PBM device	Wavelength	Fluence	Exposition time (sec)	Application protocol	Disease free survival/overall survival
Mikhailov^96^	2000	Prospective, interventional cohort	Breast cancer patients	N.S	N.S	N.S	N.S	Before surgery and in postoperative during 2 years.	PBM decreased postoperative complications by 15.3% and the duration of lymphedema. 86.9% of patients with 2^nd^ stage breast cancer and 83.3% of the patients with 3^rd^ stage breast cancer survived 10 years after PBM. 82.6% of patients with 2nd stage and 77.7% with 3rd stage breast cancer treated by PBM had no recurrences in 10 years period.
Santana‐Blank^95^	2002	Prospective, interventional cohort	Different cancer types (colon, breast, non‐Hodgkin lymphoma, lung, oral, brain, bone, gallbladder)	LD	904 nm	4.5 × 105 J/m^2^	N.S.	Daily application up to 39 months	PBM dose‐limiting toxicity was not observed. PBM is safe for clinical use and may have potential effects on Karnofsky Performance Status, antitumor activity, and quality of life, in patients with advanced cancer.
Samoilova^94^	2015	Prospective, randomized controlled trial	Breast cancer patients	LD	480–3400 nm	24 J/cm^2^	N.S.	Daily for 1 week on the sacral area	PBM may stimulate growth of human skin cells and concurrently downregulate the proliferation of tumor cells, including breast cancer cells via a systemic manner.
Antunes^92^	2017	Prospective, randomized, double‐blind, placebo controlled	Patients with squamous cell carcinoma of the head and neck	InGaAlP LD	660 nm	4 J/cm^2^	10 s/point Total 12 min	Daily from Monday to Friday, every week, immediately before RT	PBM upregulated genes related to differentiation of human epidermal keratinocytes while PBM downregulated genes associated with cytotoxicity and immune response.
Antunes^93^	2017	Prospective, randomized, double‐blind, placebo controlled	Patients with squamous cell carcinoma of the head and neck	InGaAlP LD	660 nm	4 J/cm^2^	10 s/point Total 12 min	Daily from Monday to Friday, every week, immediately before RT	Patients who underwent PBM had a statistically significant better complete response to treatment than patients in the placebo group (LG = 89.1%; PG = 67.4%; *p* = 0.013) after a median follow‐up of 41.3 months (range 0.7–101.9). Patients in the PBM group showed an increase in progression‐free survival in comparison with patients in the placebo group (61.7% vs. 40.4%; *p* = 0.030; HR: 1:93; CI 95%: 1.07–3.5) and had a tendency for better overall survival (57.4% vs. 40.4%; *p* = 0.90; HR: 1.64; CI 95%: 0.92–2.91).
Marin‐Condé^91^	2018	Prospective, randomized controlled trial	Patients diagnosed with oral or oropharyngeal SCC	LD	940 nm	83.3 J/cm^2^	360 s	N.S	There was no statistically significant difference between the PBM and control group with regard to the development of side effects
Brandão^90^	2018	Retrospective	Patients diagnosed with oral SCC	LD	660 nm	10 J/cm^2^	10 s/point 16 points in total	Daily applications for five consecutive days (Monday to Friday) throughout RT, immediately before each RT session.	The overall survival and disease‐free survival rates were 46.7 and 51.8%, respectively, after a mean follow‐up of 40.84 (± 11.71) months. 29.6% patients developed local‐regional recurrence, 6.57% patients developed distant relapse, and 12.5% patients developed new (second) primary tumors. Prophylactic PBM therapy did not alter treatment outcomes of the primary cancer, recurrence or new primary tumors, or survival.
Genot‐Klastersky^89^	2019	Retrospective	Patients diagnosed with head and neck SCC	LD	630 nm	2–3 J/cm^2^	33 s/point 6 min in total	Three times weekly up to 1 month	There was no significant difference in overall survival, time to local recurrence, and progression‐free survival, between the PBM and control patients.
Morais^88^	2020	Prospective, interventional cohort	Head and neck cancer patients	InGaAIP LD	660 nm	6.2 J/cm^2^	620 s/ session	Daily sessions (5 days per week)	The overall survival rate was 77% and disease‐free survival was 73.8%. PBM seemed to be safe in HNC patients.

Abbreviations: LD, laser diode; LED, light emitting diode; PBMT, photobiomodulation therapy.

### 3.3 In vitro studies

A total of 43 in vitro studies examining the effect of PBMT on diverse cancer cells types with varying PBM parameters were included (Table 1).^19^, ^20^, ^21^, ^22^, ^23^, ^24^, ^25^, ^26^, ^27^, ^28^, ^29^, ^30^, ^31^, ^32^, ^33^, ^34^, ^35^, ^36^, ^37^, ^38^, ^39^, ^40^, ^41^, ^42^, ^43^, ^44^, ^45^, ^46^, ^47^, ^48^, ^49^, ^50^, ^51^, ^52^, ^53^, ^54^, ^55^, ^56^, ^57^, ^58^, ^59^, ^60^, ^61^, ^62^, ^63^, ^64^

The potential for PBM to negatively influence tumor growth and/or reaction to cytotoxic treatment has had a limited evaluation to date and is currently unresolved. Conflicting data refute or support the potential for PBM to impact tumor activity and responsiveness to treatment. As noted above, given the lack of uniformity, which characterizes tumor biology, it seems probable that different tumors vary in reaction to the range of biomodulatory activities associated with PBM exposure. PBM may affect various pathways linked to negative tumor behavior, including cell proliferation and anti‐apoptosis effects. Different malignant cell lines have been used in in vitro studies to investigate the effects of PBM on cell proliferation and differentiation. They showed conflicting data by exploiting a wide diversity of PBM parameters and tumor cell lines.^48^, ^49^, ^52^, ^54^, ^61^, ^65^

Concerning the effect of PBM on malignant transformation in non‐cancer cells, an in vitro study applied PBM (660 nm, 350 mW, 15 min) during three consecutive days to epithelial cells and/or fibroblasts and no change in cell behavior was shown.^45^ Besides, in an in vitro study with normal breast epithelial cells, no malignant transformation was detected when different PBM doses and wavelengths were applied during numerous exposures.^58^

When PBM parameters were used outside the range recommended in oncology (GaAIAs laser, 809 nm, 1.96–7.84 J/cm^2^), a clear proliferation was seen in laryngeal carcinoma cells.^52^ Another study applying PBM at different wavelengths (685 and 830 nm) to Hep2 carcinoma cells, clearly showed an increase in proliferation.^55^ The differential effect of PBM on normal and cancer cells was tested in a study with osteoblasts and osteosarcoma cells. The study demonstrated that only a laser diode (830 nm) at 10 J/cm^2^ was able to increase osteoblast proliferation. On the contrary, a laser diode (780 nm) decreased osteoblast proliferation at energy densities of 1, 5, and 10 J/cm^2^. PBM did not affect osteosarcoma cells by using an 830 nm laser, while a minor proliferative effect was detected at 670 nm.^57^ In another study, human breast carcinoma and melanoma cell lines were used to investigate the effects of diverse doses of PBM at different wavelengths on cancer cells^58^: the proliferation of breast carcinoma cells increased at specific PBM doses, while numerous exposures had either no effect or reduced cell proliferation. An in vitro study on oral cancer cell lines with 1 J/cm^2 ^PBM (660 nm) showed a nonsignificant increase in the invasive potential of these cell lines.^40^ Another in vitro study in oral dysplastic and oral cancer cells suggested that PBM (660 nm or 780 nm, 40 mW, 2.05, 3.07, or 6.15 J/cm^2^) could modulate the Akt/mTOR/CyclinD1 signaling pathway linked to more aggressive cell behavior.^42^ Another report of PBM exposure of three head and neck cancer (HNC) cell lines was reported to increase the proliferation of cells in each tumor line, but not in normal tissue control.^29^

While the limits of basing broad‐reaching conclusions on in vitro assays have been noted, collectively the reports suggest it would be irresponsible to ignore the possibility that PBM could negatively impact tumor behavior. Therefore, understanding how PBM may modify tumor biology, both positively and negatively, is a research priority.^66^

Direct investigation of the radio‐modulatory effects of PBM as it affects tumor response is limited, but as with other types of cytotoxic cancer therapy, PBM may affect tumor response to radiation by the dose, fractions, and timing of PBM or radiotherapy (RT). While the data are sparse and limited to in vitro studies, the evidence suggests that PBM may act as a radiosensitizer.^30^ Another in vitro study with three HNC cell lines suggests that PBMT can enhance the sensitivity of cancer cells to chemotherapy (CT).^27^ Conversely, PBM’s reported induction of cell survival suggests a potential pathway for tumor self‐preservation.^67^ An in vitro study with oral squamous cell carcinoma (OSCC) demonstrated that PBM induced apoptosis in the absence of radiation. Moreover, PBM did not induce anti‐apoptotic effects that might stimulate tumor cell resistance to cancer therapy.^45^ When PBM (810 nm, continuous wave, 20 mW/cm^2^, 1.5 J/cm^2^) was applied to human osteosarcoma cells before NPe6‐mediated photodynamic therapy, increased apoptosis was detected as a result of a higher uptake of the photosensitizer and an increased cellular ATP.^68^ The potential enhancement of ionizing RT and CT in OSCC, was seen in PBM when applied shortly before RT and suggested that increased loco‐regional blood flow may have contributed to local tissue oxygenation, which may translate into enhanced tumor effect.^69^

Several in vitro studies demonstrated that PBM could also inhibit the proliferation of malignant cells. A study using PBM (805 nm, 4 J/cm^2^ or 20 J/cm^2^) in gingival SCC demonstrated a decrease in mitotic rate.^49^ In an in vitro study with osteosarcoma cells, PBM (830 nm) did not influence cell proliferation or protein expression.^59^ A decreased proliferation of human hepatoma cells was detected after PBM (808 nm; 5.85 and 7.8 J/cm^2^).^60^ A study with glioblastoma and astrocytoma cells showed a minor reduction in mitotic rate after PBM (805 nm and 5–20 J/cm^2^).^51^ Comparably, glioblastoma cell proliferation was inhibited by PBM (808 nm, 5 J/cm^2^).^43^ PBM at rather high cumulative doses resulted in growth inhibition of various malignant cell lines.^46^ This suggested the hypothesis that PBM may have favorable effects in the treatment of lung cancer.^70^ An in vitro study in B16F10 melanoma cells showed that high‐dose PBM (50 J/cm^2^) seemed safe, with only insignificant effects on proliferation. Furthermore, no noteworthy effect on tumor growth in a melanoma mouse model was shown. PBM at a high power density (2.5 W/cm^2^) with a very high dose of 1050 J/cm^2^ induced tumor growth in the melanoma mouse model.^62^

The wide variety of PBM parameters utilized in these studies constitutes an obstacle to arriving at meaningful conclusions, especially when the parameters are outside the scope of the MASCC/ISOO guideline that recommended PBM therapy in cancer care. Additionally, even studies using similar parameters can have differing or contradictory results. To wit, some in vitro studies suggest that PBM may favor tumor progression of oral SCC cells by activation of Akt/mTOR pathway,^42^ cellular proliferation,^29^, ^40^ and cellular migration,^28^ while other studies report a reduction in tumor growth.^25^, ^28^, ^63^ It is also important to comment that the results suggesting PBM tumor enhancement were not replicated in other studies. Generally, in vitro studies have limited applicability when compared with in vivo studies where various physiologically active cells and systems interact in the targeted tissue. There is a concomitant effect of light on endothelial, epithelial, mesenchymal, and immune cells, which must be studied together to identify real‐time effects.

### 3.4 In vivo studies

A total of 15 in vivo studies investigating the safety of PBMT in different animal cancer models were identified (Table 2).^62^, ^83^ In a study with a chemically induced OSCC hamster cheek pouch model, PBM (660 nm, 30 mW, 424 mW/cm^2^, 56.4 J/cm^2^, and 133 s, 4 J) led to tumor progression.^76^ In contrast, a study with a mouse model of multiple UV‐induced skin tumors, full‐body PBM (670 nm, twice a day, 5 J/cm^2^ for 37 days) did not enhance tumor growth in comparison with sham‐treated animals. Moreover, the tumor area slightly decreased after PBM, possibly associated with PBM‐induced antitumor immune activity or a local photodynamic effect.^77^ Similar results were seen in a rat study demonstrating that PBM was able to reduce and let even completely disappear small tumors.^82^ This led to the hypothesis that the upregulation of ATP signaling by PBM stimulated differentiation of tumor cells and apoptosis, leading to an inhibition of tumor proliferation.^84^, ^85^ A normal cell produces ATP via the process of oxidative phosphorylation. This gives a yield of around 32–38 ATPs per glucose molecule. Cancer cells naturally change from “cellular respiration” to the very ineffective glycolysis for their ATP needs (i.e. Warburg effect). Cancer cells perform anaerobic glycolysis, which implies that they produce most of their energy from glycolysis. This produces only two ATPs per glucose molecule.^86^ The potential of PBM to promote anti‐inflammatory and repair of normal tissue while not enhancing tumor cell proliferation may be related to this differential effect.

PBM was tested to stimulate hair regrowth in an animal model with leukemia, which developed chemotherapy‐induced alopecia (CIA). PBM did not alter the efficacy of CT, as 22% of the PBM‐treated rats and 20% of the control rats remained leukemia‐free.^83^ An in vivo study showed that PBM reduced the tumor growth and invasiveness in xenograft OSCC and melanoma mouse models. The authors suggested that PBM may have impacted tumor proliferation by stimulating antitumor immunity and normalizing tumor vessels.^63^ A recent study in an orthotopic animal model of head and neck squamous cell carcinoma (HNSCC) suggests that the use of PBMT does not safeguard tumor cells against the cytotoxic effect of RT.^72^

The described results indicate that diverse malignant cells may react differently to specific PBM parameters and doses. A possible explanation for this lies in the dissimilarities in the cellular microenvironment between different tumor models, as PBM has a clear effect on the cellular signal transduction pathways. The microenvironment of cancer cells differs between in vitro studies. Moreover, it is not possible to compare the microenvironment of cell culture studies with that in animal models. To improve the understanding of the dissimilarities in tumor response to PBM and how pretreatment molecular and genomic characterization of tumors can be used to establish the most appropriate PBM parameters, more in vitro and in vivo studies are needed.^87^

### 3.5 Clinical human studies

We identified nine clinical trials studying the safety of PBMT in patients with cancer, reporting disease‐free survival, overall survival, and recurrence rates (Table 3).^88^, ^89^, ^90^, ^91^, ^92^, ^93^, ^94^, ^95^, ^96^ In a prospective, randomized‐controlled trial (RCT) with HNC patients (SCC of the nasopharynx, oropharynx, and hypopharynx) undergoing CRT, PBM was used to prevent OM. The average follow‐up time was 18 months (range 10–48 months). Results showed that PBM treated patients had improved loco‐regional disease control, and a better progression‐free and overall survival.^93^ In a retrospective study with 152 advanced OSCC patients PBM (660 nm, 40 mW, 10 J/cm^2^) applied for the prevention of OM. In overall, PBM did not affect the treatment result of the primary tumor, relapse rate, development of new primary tumors, and overall survival.^90^ Similarly, a retrospective study of 222 patients with HNC who were treated with RT with or without cisplatin‐based CT investigated the safety of PBM in the management of OM. PBM did not affect the time to local recurrences, the disease‐free survival, and the overall survival.^89^

## 4 DISCUSSION

Evidence from the clinical follow‐up, and animal models and in vitro data indicate that the possible negative effect of PBM on tumor biology is not clinically relevant at the doses applied in the management of cancer therapy‐related complications. It is key to understand the effect of different PBM parameters (wavelength, fluence, energy, and time) and experimental models before applying and evaluating PBM correctly. As there is a clear contrast between in vivo and human studies versus in vitro cell culture studies, related to the tumor microenvironment.^66^, ^97^, ^98^

In vitro studies strengthen the meaning of dosimetry and exact description and control of PBM parameters for clinical, oncologic use. The benefits of the use of PBM and levels of theoretical risk should be considered for the best patient care. In our opinion, current evidence supports the use of PBM in accepted indications. The Mucositis Study Group of the MASCC/ISOO has completed a systematic review and recommended the use of PBM for management of OM in stem cell transplant and HNC patients receiving stomatotoxic cancer therapies.^6^ NICE guidelines in the UK recommend PBM also in those indications.^99^

For more than 20 years, PBM has been used in the management of OM in HNC patient and no significant adverse effects have been documented. Clinical studies have assessed tumor outcomes in patients treated with PBM.^88^, ^89^, ^90^, ^91^, ^92^, ^93^ While these clinical trial outcomes support safety in the clinical application of PBM for oral/oropharyngeal complications of cancer therapy, continuing research is warranted due to the potential diverse biological impact of PBM, and due to tumor heterogeneity and evaluating tumor behavior and overall response to therapy. Indeed tumor heterogeneity and the tumor microenvironment may have been reflected in contradictions observed in some in vitro studies. For example, 35% expressing dysregulation in the PI3K pathway, a putative site of action of PBM in OSCC.^100^ Animal and clinical trials allow assessment of the effect of PBM on the tumor and the tumor microenvironment (e.g. immune function, the surface microenvironment, and epithelial and connective tissue interactions). While additional clinical trials and follow‐up are desirable, the current evidence supports the safety of PBM in the established protocols through consensus guidelines in the management of cancer therapy‐related side effects.

### 4.1 Limitations

Due to heterogeneity of the used PBM parameters and outcome measures, a meta‐analysis of the reported data was not possible. Moreover, in this systematic review, in vitro, in vivo, and clinical trials were considered and therefore it was impractical to find a rigorous model to determine the risk of bias. This would be implemented in case the review only included clinical trials.

## 5 CONCLUSION

PBM (in the red or NIR spectrum by definition) appears safe and successful in the management of cancer therapy‐related side effects. Therefore, PBM should be considered as part of the standard of care for specific oncology purposes.^10^ Clinicians should be able to prescribe PBM by guideline recommendations using appropriate approved parameters of PBM in a clinical setting.^101^ The safety of PBM concerning the effect on the tumor response and most importantly the benefit of PBM to patients in the management of cancer treatment‐related toxicities have been shown in in vivo studies and clinical trials. Vigilance remains warranted for applications that have not been adequately documented or without guidelines due to a lack of evidence. Therefore, studies concerning the effect of PBM on malignant cell protection or enhancement of tumor growth are still needed.

The increasing number of published data in OM prevention in HNC and stem cell transplantation patients suggest that PBM does not influence tumor or treatment outcomes and overall survival. In recognition of the complexities, which govern tumor responsiveness,^102^ it remains obligatory upon the clinician to notify patients of the potential benefits and risks related to PBM. Based on the demonstrated value of PBM in supportive cancer care, continuing research may also be directed to elucidate its effect on respondents and nonrespondents to PBM, like any other treatment or mitigation modality in modern precision oncology.

## DISCLAIMER

This systematic review has been performed by an international multidisciplinary panel of clinicians and researchers, with expertise in the area of PBM. This article is informational in nature and should be used with the clear understanding that continued research and practice could result in new insights and recommendations. In no event shall the authors be liable for any decision made or action taken in reliance on the protocols included in this review paper.

## CONFLICTS OF INTEREST

The authors declare that they have no conflict of interest.

## AUTHOR CONTRIBUTION

RJB, JBE, RGN, AB, JRD, and JR conceived of the idea and the design of this systematic literature review. JR took the lead in conducting the literature search, analyzing articles, and in creating the tables and figures. RJB, JR, JBE, RGN, and JRD played a leading role in writing the manuscript. All authors discussed the results and contributed to the final manuscript. Each author has contributed in the same manner to this manuscript.
